# A comparison of the EU and US regulatory frameworks for the active substance registration of microbial biological control agents

**DOI:** 10.1002/ps.5133

**Published:** 2018-08-27

**Authors:** Coen Frederiks, Justus HH Wesseler

**Affiliations:** ^1^ Department of Social Sciences Wageningen University Brussels Belgium; ^2^ Department of Social Sciences Wageningen University Wageningen The Netherlands

**Keywords:** biopesticides, (microbial) biocontrol agents, (M)BCA, EU, USA, registration, regulation

## Abstract

**Background:**

Microbial biological control agents (MBCA) are biopesticides based on living microbes. They have huge potential for the control of pests and diseases, but have trouble reaching the European Union (EU) market. According to several authors, this is caused by the regulatory regime, which is less supportive compared with that in the USA. The main objective of this paper is to present regulatory differences between the USA and the EU, and the resulting effects and developments of registration in both regions.

**Results:**

Results show that EU registration is more complex due to differences between EU‐ and Member State (MS)‐level processes, large actor heterogeneity and low flexibility. As a result, EU registration takes, on average, ∼ 1.6 years longer than US registration. Regulatory amendments have improved EU‐level processes and led to a significant contraction of procedural time spans, but processes at the MS level have not improved and have become a larger procedural obstacle.

**Conclusion:**

The results correspond with the idea that EU registration is complex and lengthy compared with that in the USA. To improve regulation, national‐level processes should be targeted for amendment. To that end, the authors suggest various ways of expanding the registration capacity of MS. © 2018 The Authors. *Pest Management Science* published by John Wiley & Sons Ltd on behalf of Society of Chemical Industry.

## Graphical abstract

This article compares the regulation of microbial biopesticide registration in the European Union (EU) and the USA, discussing regulatory differences and resulting obstacles. EU registration takes substantially longer, lagging mostly at the national level.

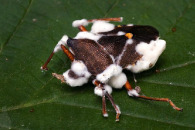

## 1 INTRODUCTION

Microbial biological control agents (MBCA) contain living micro‐organisms such as bacteria, fungi or viruses for the control of weeds or pests and diseases of crop plants, and are regulated in the European Union (EU) at both EU and Member State (MS) levels.^1^, ^2^, ^3^ MBCAs need to undergo a comprehensive risk assessment to ensure food safety. However, assessments are based on rules originally developed for synthetic pesticides and opportunities for improving risk assessment efficiency exist.^1^, ^4^

The EU assessment procedure was first laid down in Directive 91/414/EEC, in an attempt to harmonize the, until 1993, national registration schemes within the EU.^5^, ^6^ This directive was repealed by Regulation No. 1107/2009 in 2011.^3^ The amendment was designed to create regulatory circumstances that better fit the specific requirements of MBCAs^1^. With the implementation of Regulation No. 1107/2009, only 26% of registered active substances and Plant Protection Products (PPP) passed the review compared with under Directive 91/414/EEC.^7^

Market change, driven by the new regulation, created opportunities for novel pesticide products and the market share for MBCAs has grown accordingly ever since.^4^, ^8^ However, regulatory complexities lead to demanding regulatory standards. With the challenge of meeting these standards, a lack of experience, knowledge and resources in several EU or MS authorities have led to lengthy registration procedures.^1^, ^9^ As a result, relatively few MBCAs are available on the market in the EU compared with the USA.^1^, ^10^ As the largest market for MBCAs after the EU, the USA takes a different approach to MBCA registration and regulation.^7^, ^11^ Although both regions follow Organisation for Economic Co‐operation and Development (OECD) standards for risk assessment, US registration procedures are less lengthy. This has led to greater and more constant registration of MBCAs in the USA.^1^, ^11^, ^12^

Regulatory differences between the USA and the EU may pose a problem for the latter. Similar to a non‐tariff trade barrier, regulatory differences are a significant burden on international trade.^13^ In addition to hampering trade, the EU regulatory system restricts development of the MBCA sector^1^, ^9^, ^10^ and the EU's capacity for innovation.^1^, ^14^ Finally, the EU community is denied the environmental and agronomical benefits of MBCA use.^15^, ^16^ The EU regulatory framework for registration of MBCAs seems restrictive and opportunities for improvement without reducing product safety exist.^17^ The objective of this paper is to: (i) provide an overview of the EU and US regulatory frameworks for MBCA registration, (ii) determine the differences between the two regulatory frameworks including the length of approval time, (iii) present the resulting differences in terms of registration numbers and trends, and (iv) suggest possibilities for improvement.

## 2 MATERIALS AND METHODS

We determined the organization and structure of the EU framework by analysing the designated policies and relevant secondary literature. The same was done for the US framework. The resulting framework overviews allowed regulatory comparison.

To determine regional registration statistics and their developments, we derived information from EU and US online pesticide databases and related documents.^18^ The retrieved data allowed us to determine and analyse procedural time spans for all active substances that had undergone registration.

For EU registration, the procedural time span runs from the date on which an application is submitted (start of the calculation of procedural time span) to the date on which the end product is registered at a national or MS level (end of the calculation of procedural time span). All specific registration phases are considered followed by a 1‐day margin, unless specifically mentioned otherwise in EU reports. At this stage, we do not have final PPP registration dates at the MS level. Hence, it is not possible to determine procedural time spans for PPP registration at the MS level. It should be noted, therefore, that based on the maximum legal EU time frame, 27% of the entire registration timeline (i.e. active substance + PPP registration) is not included.

For US registration, the time span is considered to run from the date on which an application is submitted (start of the calculation of procedural time span) to the date on which the active substance and its end product are included in the US Federal Register (end of the calculation of procedural time span). Data are provided by the Environmental Protection Agency's (EPA) list of biopesticide active ingredients^19^, the US Federal Register and the linked federal notices and rules and Biopesticide Registration Action Documents for each active substance.^20^ We considered a reference period of January 2000 to September 2017 because this covers the most up‐to‐date available data.

## 3 RESULTS

### 3.1 EU regulatory framework

In the EU, MBCA registration is performed in two steps. During the first step, the active substance is evaluated. Data requirements for this evaluation are given in Regulation No. 283/2013 and inclusion in the list of approved active substances follows the procedures in Regulation No. 1107/2009.^3^, ^5^, ^21^ During the second step, the PPP itself is evaluated at MS level.^3^, ^4^, ^21^ The two steps do not necessarily need to be separate and subsequent: under specific circumstances, a MS can give provisional authorization of products prior to inclusion of the list for approved active substances. However, one should note that the possibility for such authorization is limited because it depends on several criteria.^3^, ^21^

#### 3.1.1 First step – evaluation of active substances at EU level

We consider three subsequent phases within active substance registration: the rapporteur Member State (RMS) phase, the risk assessment phase and the risk management phase.

**Figure 1:**
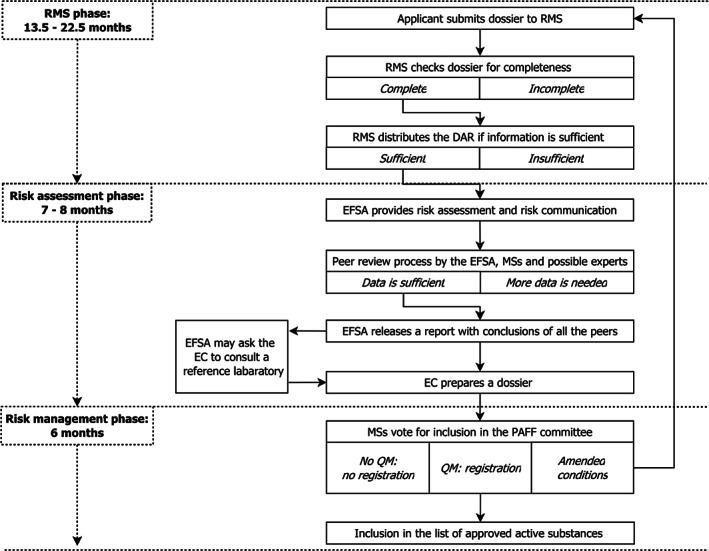
Regulatory framework for MBCA registration in the EU.

In the RMS phase, the applicant composes a dossier that contains all information on the active substance and (at least) one representative PPP. The applicant then requests registration of the active substance by delivering the dossier to a MS of its own choosing. Within 45 days, the chosen MS starts the evaluation process and this is henceforth called the designated RMS. Authorities in the RMS first check the completeness of the dossier, after which they evaluate it and subsequently distribute their Draft Assessment Report (DAR) to the other MSs, the applicant and the European Food Safety Authority (EFSA).^22^ The RMS has a maximum period of 12 months, with a possible extension of 6 months if the it decides that additional information from the applicant is required (Fig. 1).^3^

Subsequently, the EFSA provides assessments of risk and risk communication for all aspects related to food safety, during the risk assessment phase. After the EFSA has assessed the risks, the assessment undergoes a peer review process over a period of 3 months, involving all MSs and the EFSA itself. As the result of the peer review process, the EFSA releases a scientific report containing the conclusions of its peers within 4–8 months.^3^ Then, the European Commission (EC), currently represented by the Directorate General for Health and Food Safety (DG SANTE), prepares a dossier that aims at inclusion of the active ingredient into the ‘list of approved active substances’. Inclusion into the list of approved active substances implies that an active substance is eligible for use in a PPP in the EU (Fig. 1).^3^

MSs subsequently vote in the Standing Committee (SC), currently called the Standing Committee on Plant, Animals, Food and Feed (PAFF Committee)^3^, ^21^, on approving the dossier prepared by the EC. This is known as the risk management phase.^23^ Approval will only be reached by qualified majority vote, indicating that 55% of the MSs, holding at least 65% of the population, agree.^24^ After a positive risk assessment and vote session within the SC, the active substance is included in the list of approved active substances and a notice of inclusion is published in an EU official journal. Inclusion takes approximately 6 to 12 months from the date on which the dossier of the EC is presented. A ‘regular’ active substance keeps its status for 10 years. Generally qualified as ‘low‐risk’ active substances, biopesticides can be granted a 15‐year period of registration (Fig. 1).^3^, ^21^

#### 3.1.2 Second step –PPP evaluation at national level

In the second step, the PPP itself is registered at the national level. For PPP use in field crops, EU MSs are divided in three evaluation zones, coarsely linked to climatic conditions:

- Zone A – North: Denmark, Estonia, Finland, Latvia, Lithuania, and Sweden;
- Zone B – Central: Austria, Belgium, Czech Republic, Germany, Ireland, Luxembourg, Hungary, the Netherlands, Poland, Romania, Slovenia, Slovakia and the UK;
- Zone C – South: Bulgaria, Croatia, Cyprus, France, Greece, Spain, Italy, Malta and Portugal.

For use in greenhouses, post‐harvest treatments, treatment of empty storage rooms or seed treatments, the EU is considered a single zone.^3^

National registration requires that a dossier with efficacy data be submitted to a zonal rapporteur Member State (zRMS) which evaluates the product on behalf of all the MSs within its zone. All MSs in the respective zone may grant authorizations, unless their specific national conditions justify alternative conditions of use (mitigation measures) or refusal of authorization. For use in field crops, it is possible to apply for more than one zone because the zRMS should evaluate data not related to environmental and agricultural conditions. PPP applications should be evaluated by the zRMS within 12 months. If the initially submitted data do not fulfil the requirements, a maximum of 6 months additional time may be given to submit further data requested by the zRMS. If these data are not submitted on time, the application is refused. For PPPs containing an (as yet) unapproved active substance, the MS should start the evaluation after the DAR is received. Evaluation of applications for PPPs by MSs should be done within 6 months after approval of the active substance.^3^

In addition to the zonal registration procedure, mutual recognition can be applied for after authorization of the product in a first MS. If the MS where authorization was granted belongs to the same zone, mutual recognition shall be granted within 120 days. In cases in which authorization was granted by a MS or zRMS that belongs to a different zone, the authorization can be recognized by a single MS, but not for the whole zone. To ensure consistency in MS evaluations, Annex VIB of Directive 91/414/EEC provides uniform principles specific for evaluation and authorization of microbial PPPs. The same principles are also followed when active substances require re‐registration.^3^, ^5^

### 3.2 US regulatory framework

In the USA, both the PPP and its active substance are evaluated by two central authorities: the Environmental Protection Agency (EPA), which governs the active substance registration, and the Food and Drug Administration (FDA), governing the maximum residue levels (MRL).^1^, ^12^

The EPA has authority based on statutes within the Federal Food, Drug and Cosmetic Act (FFDCA 1938) and the Federal Insecticide, Fungicide and Rodenticide Act (FIFRA 1947).^11^, ^12^ In addition, the Food Quality Protection Act (FQPA 1996) sets further standards for new and old pesticides, making requirements regarding processed and unprocessed foods more uniform.^12^ Finally, the Pesticide Registration Improvement Act (PRIA) established specific fees and specific timelines for different types of pesticide registration actions that may vary between 4 and 18 months. There have been three versions of the PRIA: PRIA 1, PRIA 2 (renewal) and PRIA 3 (extension), implemented in 2004, 2007 and 2012 respectively.^22^ Biological control agent (BCA) data requirements are listed in Title 40 of the Code of Federal Regulations (40 CFR) Part 158 and more specifically, data requirements for MBCA are listed in Subpart V: Microbial Pesticides 40 CFR 158.2100 through 40 CFR 158.2174.^25^ The EPA also published guidelines and data requirements that need to be fulfilled to support registration. These may include the Office of Chemical Safety and Pollution Prevention (OSCPP) series 830, 850, 870 and 885.^26^, ^27^, ^28^, ^29^ Prior to the formal start of the procedure, an applicant may approach the EPA in a pre‐submission meeting. Although not required, these meetings are recommended by the EPA. In these meetings, applicants are advised what studies are necessary for the product up for submission. These studies depend on preliminary identification of the product and the amount of data available from the literature or other sources. The applicant then submits a summary of the meeting(s) to the agency to receive comments and confirmation of completeness.^21^, ^30^

**Figure 2:**
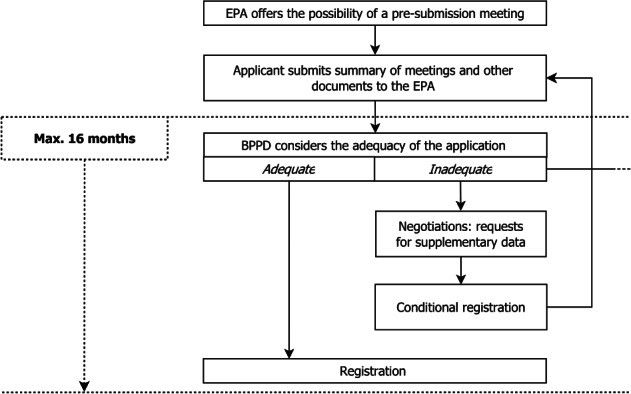
Regulatory framework for MBCA registration in the USA.

Following the optional pre‐submission meetings, the applicant must undertake three steps when processing an application to determine whether the application is complete and contains sufficient information for the EPA to make a regulatory decision. First, the EPA checks whether the application is complete enough to be assigned to a division for review in the initial screen for completeness, which takes 21 days. Second, a preliminary technical screen is done to determine if the data are (i) accurate and complete, (ii) consistent with proposed labelling and any tolerance and tolerance exemption, such that (iii) subject to full review, could result in the granting of the application. If information is not sufficient in the second step, the applicant has 10 business days to provide the required information. Failure to comply with the response period results in rejection of the application.^25^ After receiving the meeting summaries, the Biopesticides and Pollution Prevention Division (BPPD) has a maximum of 19 months from receipt of a complete application to the registration decision according to the PRIA 3 timelines.^12^, ^25^, ^30^ A registration decision may result in registration, renegotiation due to inadequacies, or a full rejection (Fig. 2).^25^

If data are missing or classified as ‘supplementary’, risk is low enough to market the product, or there is any other reason to be flexible, the US framework may allow for conditional registration in the form of: (i) emergency exemptions, or (ii) state‐specific registrations. A registration is valid for 15 years throughout the USA.^21^, ^25^

In terms of finance, the US has some special regulations. First, the US Department of Agriculture (USDA) may offer grants for registration‐related research. The USDA does this through Inter‐Regional Research Project Number 4, an initiative to support of the registration of minor use pesticides.^31^ Second, small and medium‐sized enterprises (SMEs) may be funded through the ‘Small Business Innovative Research program’^11^ In addition to these financial advantages, the USA may provide financial exemptions to SME or government bodies, which are often exempted from EPA reviewing fees.^21^

The US framework also allows for an exemption of registration in the case of minimum risk pesticides. All MBCAs placed on the EPA's 25b list, which is found under 40 CFR 158.25(f), and their active substances are exempted from federal registration under certain conditions.^27^, ^28^ It should, however, be noted that states may not agree with the EPA's 25b list, and this may still lead to mandatory registration at US Federal State level.^32^

### 3.3 Exemptions and waivers EU and USA

In the USA, certain data requirements may be met with a ‘waiver’ that, if accepted, allows the applicant to not provide certain studies that are normally required by the OCSPP guidelines. The applicant has to apply for a waiver based on published literature or by providing their own data.^11^, ^25^, ^30^ The waiver system does not exist formally in the EU, however, EU applicants may provide a scientifically reasoned justification for not providing certain parts of the registration dossiers.^3^ Formal data waivers in the USA are accepted more easily than a reasoned case in the EU.^21^

### 3.4 Overall comparison EU and US regulatory frameworks

The US regulatory framework is less complex than that used by the EU in many ways. In the EU, more authorities are involved: EU‐level processes are run by four major authorities, whereas there are only two in the USA. In addition to EU‐level processes, national registration requires MS authorization. This creates a heterogeneous procedure in the EU, leading to several hurdles to registration (Table 1).

**Table 1:** Overview of the framework comparison between the EU and the USA. Source: author's elaboration

Aspect	EU regulatory framework		US regulatory framework
Regulation	Regulation No. 1107/2009		40 CFR Part 158
	Regulation No. 283/2013		
Regulation type	Based on chemical pesticides		Accustomed to biopesticides
Guidelines	None		OSCPP Series 830, 850, 870 or 885
Procedural time span	EU (AS only)	EU ± MS (Incl. PPP)	Max. 7 months (experimental use permit)
	Max. 26.5–47.5 months	Max. 59.5–65.5 months	Max. 18 months (regular)
Registration period	10 years		15 years
	15 years (low‐risk AS)		
Authorities involved	RMS		EPA‐BPPD
	EC‐DG SANCO		FDA
	EFSA		
	SCFCAH		
	zRMS (national PPP registration)		
Barriers	Long‐lasting procedural time span		
	Multiple RMSs: differ in expertise		
	National registration still a hurdle		

### 3.5 Developments EU and US registration

**Figure 3:**
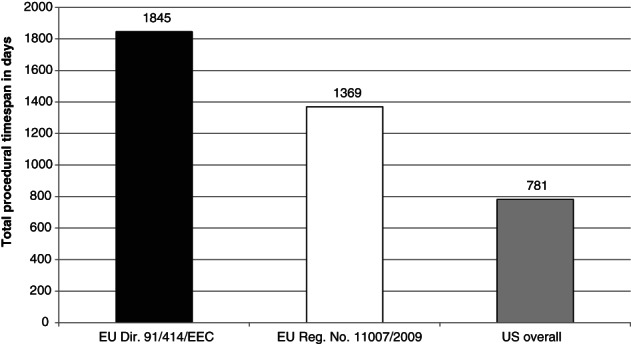
Average time span in days for the USA and under EU Regulation No. 1107/2009 and Directive 91/414/EEC.

Since January 2000, 47 MBCAs have been registered in the EU and 73 in the USA (Appendices 1 and 2); of these, 13 have been registered both in the EU and the USA. Some 34 of the MBCAs registered in the EU were registered prior to the reforms in 2009 and 14 since. On average, approval took 1678 days in the EU. The average procedural time for active substance registration decreased by 476 days with the implementation of Regulation No. 11007/2009. Average PPP registration takes 629 days.^33^ In the USA, the average procedural time is 588 days less than EU registration under Regulation No. 11007/2009 (Fig. 3).

**Figure 4:**
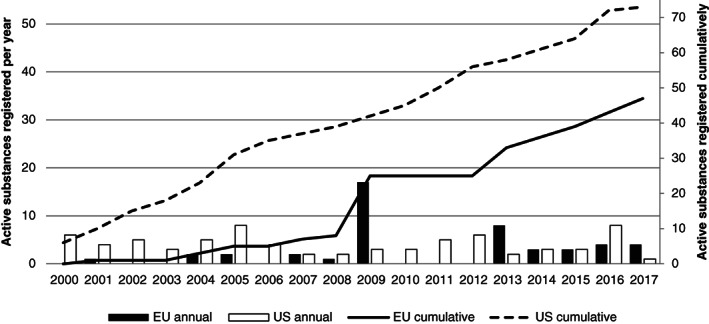
Annual and cumulative numbers of active substance registrations in the EU and USA.

Beginning with the first harmonized EU registration in 2001, the EU has shown modest and irregular registration of just under two active substances during the first 8 years. In 2009, the EU's list of approved active substances was expanded by 17 re‐registered active substances (already on the EU market under the former national market registration).^34^ The 2009 peak thus does not show actual net expansion of the EU's list of approved active substances or a potential market for MBCAs. Since 2013, implementation of Regulation No. 1107/2009 has seemed to bear fruit because the cumulative number of registrations has increased steadily at a rate of more than four active substances per year. In the USA, annual registration of new active substances is more constant: the registration rate has been an approximately four active substances per year throughout the reference period (Fig. 4).

#### 3.5.1 Development first step – active substance registration on the EU level

To analyse the procedural time for registration, 31 observations (applications) were available. Observation data are pulled from specific DG Sante review documents. The observations include new and successful registrations only (i.e. exclude registration reviews and non‐approved active substances). The oldest observation dates from January 2001 and the most recent from March 2017. With a minimum of 1103 days and a maximum of 4159 days, the observed procedural time spans show a maximum difference of 3056 days. Although the mean time span is 2109 days, the median at 2116 days exceeds that.

Appendix A gives a time span overview of EU registration cases, running from the date of application to the date of approval within the reference period 2000–2017. Time spans vary substantially: documentation shows cases of > 11 years to recent cases with a procedural time of ∼ 3 years. Procedural time spans such as that for *Spodoptera exigua* nuclear polyhedrosis virus (11.4 years) or *Pseudomonas chlororaphis* strain MA342 (9.8 years) were mainly caused by inexperience with the, at that time, novel integrated EU approach to active substance registration.^21^ This inexperience caused uncertainty about what data to collect or submit and led to a particular lengthy RMS phase.^34^ Procedural time spans seem to contract over time.

Analysis confirms a negative correlation between the procedural time span and date of application. A linear regression analysis for this relation, including regulatory amendment as an extra variable, shows that both variables have a significant negative influence on procedural time span. The outcomes of the analysis allow for an estimation of the trends in procedural time spans through a linear function (1) (Table 2).

(1) $$Y_{i}=\alpha +\beta X_{t}+D_{\mathrm{Reg}.\;1107/2009}+\epsilon_{i}$$

**Table 2:** Multiple regression output for the procedural time span of active substance registration in days over time and under regulatory amendments in the EU and the USA

	Coefficient	SE†	*P*‐value
EU overall			
Intercept	3194.676	193.960	0.000
Days since first application*	−0.181	0.054	0.002
Regulation No. 1107/2009	−632.302	247.401	0.016
EU Directive 91/414/EEC			
Intercept	3200.600	247.619	0.000
Days since first application*	−0.183	0.070	0.018
EU Regulation No. 1107/2009			
Intercept	2267.353	870.808	0.025
Days since first application*	−0.136	0.132	0.323
US overall			
Intercept	974.604	95.514	0.000
Days since first application*	−0.65	0.026	0.016

* Slope of the function, change in procedural time span over time (days since first application)

† Standard error

The linear model represents the procedural time span for active substance registration in days. Denoted by *Y*_*i*_, procedural time span is the dependent variable. The independent variable is the date the registration procedure started, and is given by the number of days since the first application and denoted by *βX*_*t*_. Regulatory change due to the shift from Directive 414/91 EEC to Regulation No. 1107/2009 is denoted by a dummy variable *D*_Reg. 1107/2009_. The dummy variable takes into account the effect of regulatory reform. The intercept, α, represents the initial time in days. Values for the regression model imply that the estimated procedural time span on the first day of the reference period (*t*_0_) is 3195 days. From that moment, each subsequent day on the timeline results in a 0.181‐day decrease in the procedural time span. The qualitative coefficient ‘Regulation No. 1107/2009’ implies that, on average, the procedural time span has decreased by 632 days under Regulation No. 1107/2009 compared with the average under Directive 91/414/EEC (Table 2).

**Figure 5:**
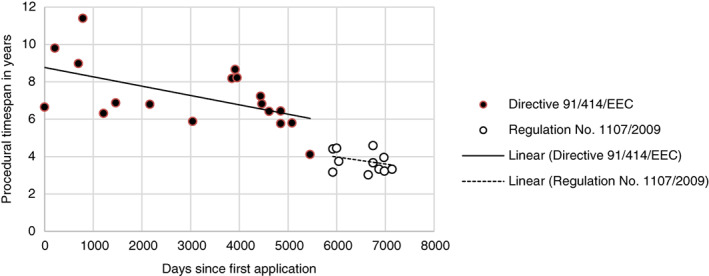
Procedural time span of EU registration under Directive 91/414/EEC and Regulation No. 1107/2009 plotted against the number of days since the first application.

The procedural time span in the EU thus declined steadily under Directive 91/414/EEC. After implementation of Regulation No. 1107/2009, the time span underwent a further sudden decrease (Fig. 5).

**Figure 6:**
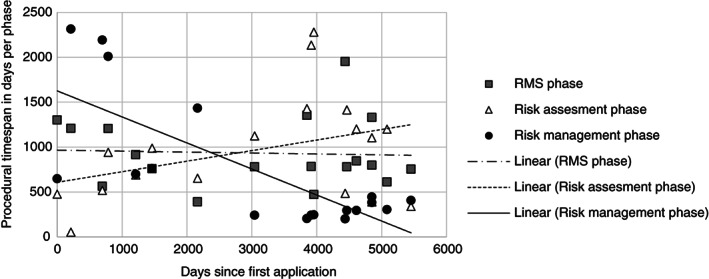
Procedural time span of the rapporteur Member State (RMS), risk assessment and risk management phase in days plotted against the number of days since first the application under Directive 91/414/EEC.

**Figure 7:**
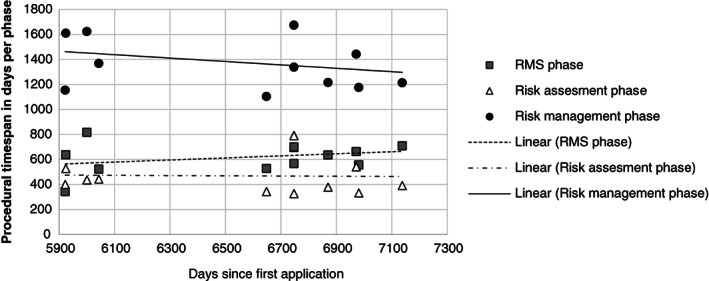
Procedural time span of the rapporteur Member State (RMS), risk assessment and risk management phase in days plotted against the number of days since first the application under Regulation No. 1107/2009.

Separate regression analyses for Regulation No. 1107/2009 and Directive 91/414/EEC show that an active substance registered under Regulation No. 1107/2009 and at time *t*_0_ (1 October 2013), would be registered 933 days faster than an active substance under registered under Directive 91/414/EEC and at time *t*_0_ (7 January 2001) would have been. The significant daily decline under Directive 91/414/EEC is caused by contraction of the risk management phase: the RMS phase remains roughly the same and the risk assessment phase increases under Directive 91/414/EEC (Fig. 6). The lower daily decline under Regulation No. 1107/2009 seems to be caused by contraction of both the risk assessment and risk management phases (Fig. 7). However, given the limited number of observations for Regulation No. 1107/2009, this cannot yet be considered significant (Table 2).

#### 3.5.2 Development of EU active substance registration broken down into phases

**Figure 8:**
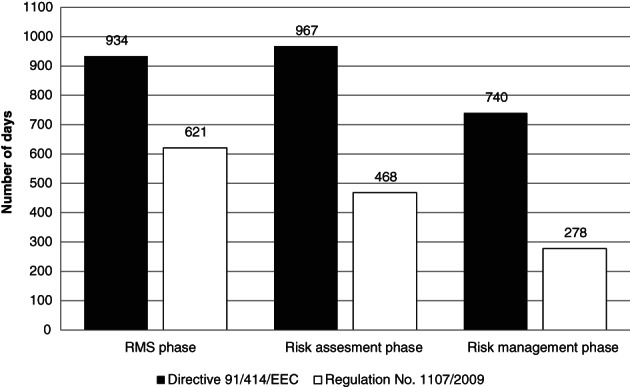
Average number of days per phase of EU active substance registration under Directive 91/414/EEC and Regulation No. 1107/2009.

After implementation of Regulation No. 1107/2009, the RMS phase decreased by 33.5%, the risk assessment phase decreased by 51.6% and the risk management phase decreased by 62.5% (Fig. 8). Overall, the average procedural time span decreased by 48.2%.

Only 11 MSs performed an RMS between 2000 and 2017. Under Directive 91/414/EEC, Sweden, Italy and Estonia have been the most encouraging RMSs, with a low average time span. The UK was the least encouraging RMS; this can be explained by one exceptionally lengthy registration case. As the second slowest performer, longer RMS time spans were more common in the Netherlands. This might have been due to a lack of resources and experience, especially as the Netherlands was RMS for four of the five ‘first‐ever’ active substances.

Following reform, RMS time spans decreased in general. Belgium, Germany, France and the Netherlands are the only ones to have yet performed an RMS under Regulation No. 1107/2009. Germany being an exception, reform led to France, Belgium and the Netherlands becoming the three most encouraging RMS candidates in terms of the average time span (Fig. 10).

**Figure 9:**
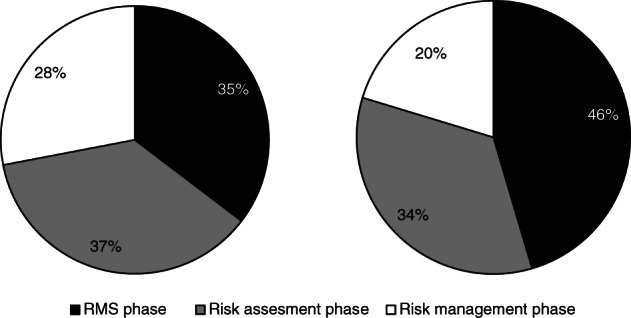
Relative build‐up of procedural time span for active substance registration in the EU before (left) and after (right) the implementation of Regulation No. 1107/2009.

**Figure 10:**
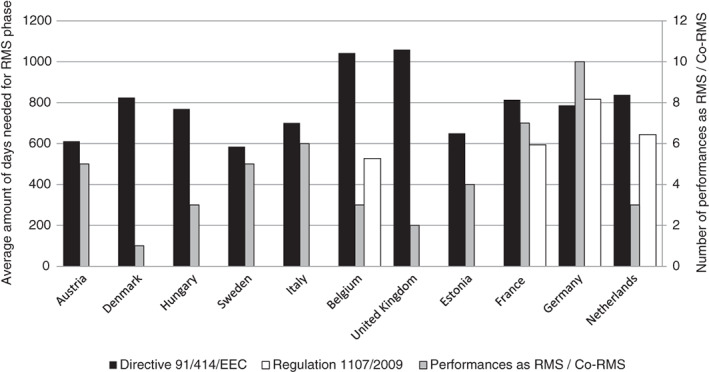
Number of performances as rapporteur Member State (RMS) per Member State and the average number of days needed as RMS per member State under Directive 91/414/EEC and Regulation No. 1107/2009.

#### 3.5.3 Development second step – PPP registration on the national level

On average, PPP registration took 629 days between 2013 to 2015. In 2013 and 2014, four of five zRMS procedures exceeded the procedural deadlines, leading to legal compliance for only 21%. For subsequent approval of the efficacy report by other MSs in the designated zone, all decisions exceeded procedural deadlines and only 15% were legally compliant. Finally, mutual recognition exceeded deadlines in five of seven cases, leading to a legal compliance of 29%. Because of these delays in PPP registration, the EU is witnessing an increasing number of emergency registrations, although mainly for inorganic active substances.^35^

With implementation of Regulation No. 1107/2009, the proportions of the three phases within the total procedure changed. The RMS phase increased, whereas the risk management phase decreased. This caused the RMS phase to become a relative bottleneck following regulatory reform (Fig. 9).

#### 3.5.4 Development of overall US registration

To analyse the procedural time span for US registration, 62 observations were available. Data for observations were pulled from rules, notices and supporting material from the Federal Register. The observations include initial successful registrations only. This also concerns two cases that are subsequent to an Experimental Use Permit. The oldest observation dates from December 2001 and the newest from June 2017. With a minimum of 51 days and a maximum of 2060 days, the observed procedural time spans have a maximum difference of 2009 days. Although the mean is 778 days, the median is 683 days.

Appendix B presents time spans in the USA. Lengthy cases may be caused by joint registrations for both the US EPA and the Canadian Pest Management Regulatory Authority (PMRA) (*e.g. Chondrostereum purpureum* strain HQ1), others are caused by submitting insufficient dossiers (e.g. *Vertillicum* isolate WCS 850). However, due to missing documentation in the Federal Register (i.e. registration actions documents or Federal notices), not all outliers can be explained. Procedural time spans seem to decrease slightly over time.

**Figure 11:**
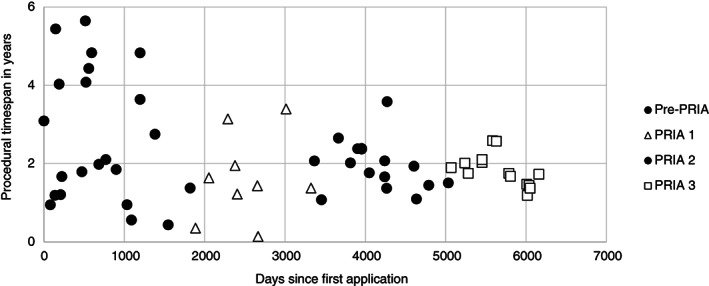
Procedural time span of US registration under subsequent versions of the Pesticide Registration Improvement Act (PRIA) plotted against the number of days since the first application.

Analysis confirms an overall negative correlation between the procedural time span and date of application in the USA. Since the PRIA 1 came into force in 2004,^22^ the maximum length of the US registration procedure has become more consistent. Implementation of PRIA 2 and PRIA 3 seems to have further contributed to this trend (Fig. 11).^33^ Regression analysis does not show a significant effect for PRIA amendments as a variable. Regression analysis for the procedural time span in days (dependent) and days since the first US registration (independent) within the reference period (2000–2017) shows a significant negative relation between procedural time span and date of application (Table 2).

The model developed through linear regression again represents the procedural time span for active substance registration in days (Equation 1). The variables are the same as for the EU, but for the USA the dummy variable for Regulation No. 1107/2009 is omitted. The values show that the estimated procedural time span on the start date of the reference period (*t*_0_) is 974 days. From that moment, each subsequent day on the timeline results in a 0.065‐day decrease in the procedural time span (Table 2).

### 3.6 EU versus US developments

**Figure 12:**
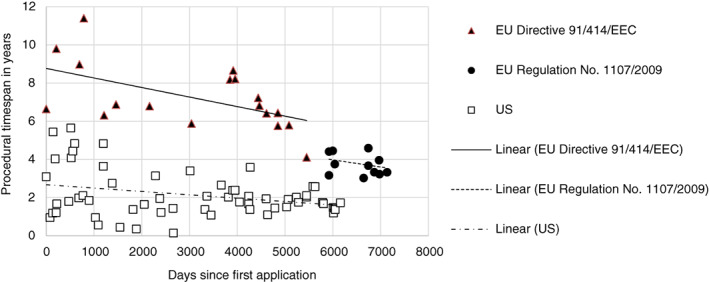
Procedural time span of overall US registration and EU registration under Directive 91/41/EEC and Regulation No. 1107/2009 plotted against the number of days since the first application.

Based on analyses of the registration procedure in both regions, estimations show a significant trend of a decrease in procedural time span for active substance registration. Although the procedural time span remains substantially shorter in the USA, the gap between the EU and the USA has become substantially less due to daily contraction under Directive 91/414 EEC and the sudden contraction driven by implementation of Regulation No. 1107/2009 (Fig. 12).

### 3.7 Same active substances, different fates

**Figure 13:**
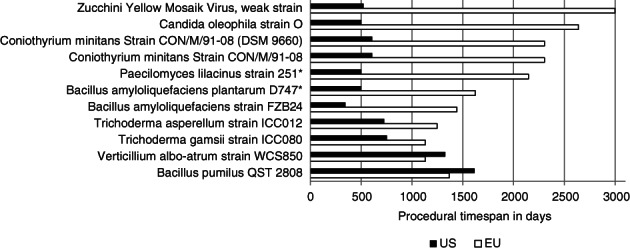
Comparison of time span in days for active substances registered in both the EU and USA, cases marked with an asterisk started registration in the EU first.

Thirteen active substances have been registered in both the EU and the USA, of which 11 can be compared based on their documentation. The difference in procedural time span between the EU and the USA varies substantially. With a procedural time span of 196 and 249 days less than the procedural timespan in the US, registration of *Verticillium albo‐atrum* strain WCS850 and *Bacillus pumilus* QST 2808, respectively, were registered the quickest in the EU. With an additional 2475 days in the EU, the case for zucchini yellow mosaic virus shows the largest difference in procedural time span (Fig. 13). Despite these already substantial differences, it should be noted that the US time span includes PPP registration, whereas the EU time span includes active substance registration only. Two active substances were registered in the EU first and on average, US registrations were completed 1269 days quicker.

A major proportion of the EU's protracted procedural time span is caused by protracted RMS phases. In the case of zucchini yellow mosaic virus, the applicant failed to supply supplemental information to the EFSA.^36^ For the registration of *Candida oleophila* strain O, *Coniothyrium minintans* and *Bacillus amyloliquefaciens,* requests for supplementary studies caused the RMS phase to be lengthy.^37^, ^38^, ^39^ In the case of *Paecilomyces lilanicus*, protraction was due to both the RMS phase and the need for expert consultation in the peer review phase.^40^

## 4 DISCUSSION

The MBCA registration procedure in the EU seems substantially slower than procedures in the USA, taking an additional 1.62 years (43%) on average. The EPA's upfront screening process tends to deny some applications at the outset. This has a positive effect on procedural time, but is not captured in the data. Nevertheless, the calculated average delay in registration leads to foregone benefits of using the MBCA and thus to costs due to the delay. Benjamin *et al*. show that the (foregone) socio‐economic benefits of biological control of European corn rootworm in potato and maize might be €48.7 million annually for France, Italy, Spain, Germany and Romania combined.^41^ Although the costs of delay depend on many factors and vary per MBCA, this gives an indication of the economic importance of the EU's delay in registration compared with the USA.

When looking at an almost similar EU process such as approval for genetically modified organism (GMO) techniques, we see a delay of 1.93 years (39.9%) in the EU compared with the USA.^23^ The GMO approval process is delayed mostly due to a MS voting gridlock.^42^ Given the absence of such a problem in the MBCA registration process, one can reason that there is potential for the time span of the MBCA registration process in the EU to decrease further.

Both the sudden contraction in 2009 and the subsequent continuous contraction in the procedural time span for all EU‐level processes (risk management phase and risk assessment phase) show that implementation of Regulation No. 1107/2009 is paying off in this regard. The contraction is likely to be further supported by a growing demand for organic products^8^, ^43^ and societal pressure to move towards a more sustainable mode of food production.^44^ By gaining more experience, it is also likely that increased efficiency in risk assessment and management will contribute to the continuous contraction of the procedural time span.^45^

Although the time span for EU‐level processes did improve, MS‐level processes are still lagging, suggesting that this is where the EU can gain in terms of efficiency. Adding to that, Zilberman and Wesseler show that the economic importance of the first 2 years of the procedure is greater than that of subsequent years.^46^ This is interesting in the context of EU registration, because the RMS phase (first phase, ± 1.5 years) is more of a bottleneck since implementing Regulation No. 1107/2009: EFSA review documents in the EU pesticide database show that five of nine RMS cases exceeded their deadline between 2009 and 2016. Streamlining the RMS phase should therefore be a focus for improving of EU procedures. Because RMSs with a designated evaluation authority (UK, France, Sweden, the Netherlands) tend to be more efficient due to the swifter accumulation of relevant experience^6^, an improvement strategy could be to restrict RMS participation to these MSs. Another strategy could be to appoint certain cases to RMSs with experience within a specific category (e.g. related to target pest/disease, or crop).

In addition to the RMS phase, PPP registration poses another obstacle at the MS level. To date, stricter guidelines related to deadlines at the MS level have not been successful.^47^ The remaining low levels of regulatory compliance suggest that the EU should therefore act within its mandate rather than expand MS registration capacity by addressing the lack of resources, infrastructure or experience. This can be done via exchange with the EFSA or successful RMSs such as Belgium, France and the Netherlands. As one of these strong performers, the Netherlands provided an example of how to expand capacity for registration of biopesticides through the so‐called ‘Green Deal Project’, a 3‐year project in which the Dutch government worked on improved national BCA registration together with key public and private stakeholders. Outcomes and follow‐ups focused on not only capacity improvement, but also new legislative forms that enable higher success ratios for low‐risk active substances and PPP (through, for example, waivers and financial support measures).^48^

## 5 CONCLUSION

The EU regulatory framework for pesticide active substance registration governs all types of pesticides (i.e. both chemical and organic). The procedure has two steps; first, active substance registration at the EU level and second, PPP registration at a MS level. On average, both steps combined take 65.7 months under Regulation No. 1107/2009. By contrast to the EU framework, the US framework is accustomed to biopesticides. Furthermore, the PPP and active substance are evaluated simultaneously. On average, US registration takes 25.7 months. The US procedure is more flexible: it is less heterogeneous, involves a smaller range of actors and takes less time, and trumps the EU system through data ‘waivers’, financial exemptions and conditional registrations.

The result of the initial regulatory discrepancies between the two regions is that, between 2000 and 2005, the number of active substances registered under harmonized EU regulation lagged compared with the USA. But US numbers increased only slightly after 2005 and, since regulatory reform in 2009, EU registrations have been increasing. Although both regions showed a steady and significant decrease in the procedural time span between 2000 and 2016, the decrease was strongest in the EU, causing the gap between the two regions to decrease. Under Directive 91/414/EEC, the EU procedural time span decreased significantly over time. After implementation of Regulation No. 1107/2009, the procedural time span showed another significant and sudden (i.e. immediate) decrease. The amendment caused all three phases of active substance registration to contract, but the RMS phase has become the greater obstacle. Having an experienced and well‐performing RMS has therefore become more important. With the majority of MSs failing to comply with regulatory standards and delaying registration, PPP registration has become another important obstacle. Processes on the MS level thus seem to be the greatest bottleneck and should be prioritized by the EU.

Given the limited number of observations, we analysed registration by applying linear models. However, because the MBCA market is diverse and complex, registration trends will likely depend on more than just time and regulatory amendments. Factors might, for example, include the regulator's preference or bias in prioritizing certain cases (based on, for example, complexity or familiarity), the origin of an applicant or other regulatory amendments. To account for such non‐linearities, future research should consider multivariate regressions to control for compositional effects. For a comprehensive approach, such analysis should also be performed for PPP registration data.

In addition to analysing registration itself, it would be interesting to determine what the current regulatory framework entails for the EU economically. A suggestion for future research is to use the results in this study and attempt to determine the cost of the EU's procedure compared with, for example, the US system. As has been done for the introduction of vitamin A‐enriched rice in India, Wesseler's and Zilberman's calculation of a government's or regulator's ‘perceived costs’ could serve as a method to express a regulatory regime financially.^46^ These quantified results could then be used to target or prioritize parts of a regulatory framework and its possible regulatory amendments.

## Appendix

**Table:** **Appendix A.** Overview of the considered active substances in the EU within the reference period (2000–2017)

Active substance	Year first registered	RMS phase		Risk assessment phase		Risk management phase		Time span in days	Regulatory framework
*Paecilomyces fumosoroseus* ‘PFR 97’	2001	18‐5‐1994	9‐12‐1997	10‐12‐1997	31‐3‐1999	1‐4‐1999	27‐4‐2001	2426	Directive 91/414/EEC
*Pseudomonas chlororaphis* strain MA342	2004	15‐12‐1994	7‐4‐1998	8‐4‐1998	31‐5‐1998	1‐6‐1998	30‐3‐2004	3578	Directive 91/414/EEC
*Ampelomyces quisqualis* strain AQ10	2005	12‐4‐1996	28‐10‐1997	29‐10‐1997	31‐3‐1999	1‐4‐1999	8‐10‐2004	3276	Directive 91/414/EEC
*Spodoptera exigua* nuclear polyhedrosis virus	2007	12‐7‐1996	1‐11‐1999	2‐11‐1999	31‐5‐2002	1‐6‐2002	15‐5‐2007	4159	Directive 91/414/EEC
*Coniothyrium minitans* strain CON/M/91‐2008 (DSM 9660)	2004	10‐9‐1997	13‐3‐2000	14‐3‐2000	1‐2‐2002	2‐2‐2002	4‐7‐2003	2304	Directive 91/414/EEC
*Gliocladium catenulatum* strain J1446	2005	19‐5‐1998	15‐6‐2000	16‐6‐2000	28‐2‐2003	1‐3‐2003	8‐10‐2004	2509	Directive 91/414/EEC
*Bacillus subtilis* str. QST 713	2007	19‐4‐2000	15‐5‐2001	16‐5‐2001	28‐2‐2003	1‐3‐2003	14‐7‐2006	2479	Directive 91/414/EEC
*Paecilomyces lilacinus* strain 251	2008	15‐9‐2002	3‐11‐2004	4‐11‐2004	3‐12‐2007	4‐12‐2007	22‐1‐2008	2147	Directive 91/414/EEC
*Adoxophyes orana* GV strain BV‐0001	2013	29‐11‐2004	13‐8‐2008	14‐8‐2008	12‐7‐12	12‐7‐12	13‐7‐12	2986	Directive 91/414/EEC
*Paecilomyces fumosoroseus* strain Fe9901	2013	4‐2‐2005	29‐3‐2007	30‐3‐2007	31‐1‐2013	1‐2‐2013	15‐3‐2013	3161	Directive 91/414/EEC
Zucchini Yellow Mosaic Virus, weak strain	2013	16‐3‐2005	30‐6‐2006	1‐7‐2006	27‐9‐2012	28‐9‐2012	20‐11‐2012	2999	Directive 91/414/EEC
*Bacillus thuringiensis* subsp. *aizawai* strains ABTS‐1857 and GC‐91	2009	30‐11‐2005	1‐11‐2007	2‐11‐2007	10‐7‐2008	11‐7‐2008	11‐7‐2008	1248	Directive 91/414/EEC
*Bacillus thuringiensis* subsp. *israeliensis* (serotype H‐2014) strain AM65‐52	2009	30‐11‐2005	1‐11‐2007	2‐11‐2007	10‐7‐2008	11‐7‐2008	11‐7‐2008	1248	Directive 91/414/EEC
*Bacillus thuringiensis* subsp. *kurstaki* strains ABTS 351, PB 54, SA 11, SA12 and EG 2348	2009	30‐11‐2005	1‐11‐2007	2‐11‐2007	10‐7‐2008	11‐7‐2008	11‐7‐2008	1248	Directive 91/414/EEC
*Bacillus thuringiensis* subsp. *tenebrionis* strain NB 176 (TM 14 1)	2009	30‐11‐2005	1‐11‐2007	2‐11‐2007	10‐7‐2008	11‐7‐2008	11‐7‐2008	1248	Directive 91/414/EEC
*Beauveria bassiana* strains ATCC 74040 and GHA	2009	30‐11‐2005	1‐11‐2007	2‐11‐2007	10‐7‐2008	11‐7‐2008	11‐7‐2008	1248	Directive 91/414/EEC
*Cydia pomonella* Granulovirus (CpGV)	2009	30‐11‐2005	1‐11‐2007	2‐11‐2007	10‐7‐2008	11‐7‐2008	11‐7‐2008	1248	Directive 91/414/EEC
*Lecanicillium muscarium* (formerly *Verticillium lecanii*) strain Ve6	2009	30‐11‐2005	1‐7‐2007	2‐7‐2007	10‐7‐2008	11‐7‐2008	11‐7‐2008	1248	Directive 91/414/EEC
*Metarhizium anisopliae* var. *anisopliae* strain BIPESCO 5/F52	2009	30‐11‐2005	1‐7‐2007	2‐7‐2007	10‐7‐2008	11‐7‐2008	11‐7‐2008	1248	Directive 91/414/EEC
*Phlebiopsis gigantea* (several strains)	2009	30‐11‐2005	1‐4‐2007	2‐4‐2007	10‐7‐2008	11‐7‐2008	11‐7‐2008	1248	Directive 91/414/EEC
*Pythium oligandrum* M1	2009	30‐11‐2005	1‐6‐2007	2‐6‐2007	10‐7‐2008	11‐7‐2008	11‐7‐2008	1248	Directive 91/414/EEC
*Streptomyces* K61 (formerly *S. griseoviridis*)	2009	30‐11‐2005	1‐4‐2007	2‐4‐2007	10‐7‐2008	11‐7‐2008	11‐7‐2008	1248	Directive 91/414/EEC
*Trichoderma asperellum* (formerly *T. harzianum*) strains ICC012, T25 and TV1	2009	30‐11‐2005	1‐6‐2007	2‐6‐2007	10‐7‐2008	11‐7‐2008	11‐7‐2008	1248	Directive 91/414/EEC
*Trichoderma atroviride* (formerly *T. harzianum*) strains IMI 206040 and T11	2009	30‐11‐2005	1‐7‐2007	2‐7‐2007	10‐7‐2008	11‐7‐2008	11‐7‐2008	1248	Directive 91/414/EEC
*Trichoderma gamsii* (formerly *T. viride*) strain ICC080	2009	30‐11‐2005	1‐6‐2007	2‐6‐2007	10‐7‐2008	11‐7‐2008	11‐7‐2008	1248	Directive 91/414/EEC
*Trichoderma harzianum* strains T‐22 and ITEM 908	2009	30‐11‐2005	1‐7‐2007	2‐7‐2007	10‐7‐2008	11‐7‐2008	11‐7‐2008	1248	Directive 91/414/EEC
*Trichoderma polysporum* strain IMI 206039	2009	30‐11‐2005	1‐10‐2007	2‐10‐2007	10‐7‐2008	11‐7‐2008	11‐7‐2008	1248	Directive 91/414/EEC
*Verticillium albo‐atrum* (formerly *Verticillium dahliae*) strain WCS850	2009	30‐11‐2005	1‐7‐2007	2‐7‐2007	10‐7‐2008	11‐7‐2008	11‐7‐2008	1248	Directive 91/414/EEC
*Candida oleophila* strain O	2013	12‐7‐2006	15‐11‐2011	16‐11‐2011	14‐3‐2013	14‐3‐2013	15‐3‐2013	2638	Directive 91/414/EEC
*Helicoverpa armigera* nucleopolyhedrovirus (HearNPV)	2013	7‐8‐2006	26‐9‐2008	27‐9‐2008	10‐8‐2012	11‐8‐2012	15‐5‐2013	2490	Directive 91/414/EEC
*Spodoptera littoralis* nucleopolyhedrovirus	2013	2‐1‐2007	26‐4‐2009	27‐4‐2009	10‐8‐2012	11‐8‐2012	15‐5‐2013	2342	Directive 91/414/EEC
*Trichoderma atroviride* strain I‐1237	2013	28‐8‐2007	19‐4‐2011	20‐4‐2011	14‐5‐2012	15‐5‐2012	20‐11‐2012	2104	Directive 91/414/EEC
*Pseudomonas* sp. strain DSMZ 13134	2014	28‐8‐2007	3‐11‐2009	4‐11‐2009	12‐11‐2012	13‐11‐2012	16‐6‐2013	2349	Directive 91/414/EEC
*Aureobasidium pullulans* (strains DSM 14940 and DSM 14941)	2014	17‐4‐2008	19‐12‐2009	20‐12‐2009	2‐4‐2013	3‐4‐2013	16‐7‐2013	2116	Directive 91/414/EEC
*Trichoderma asperellum* (strain T34)	2013	22‐4‐2009	16‐5‐2011	17‐5‐2011	20‐4‐2012	21‐4‐2012	20‐11‐2012	1501	Directive 91/414/EEC
*Bacillus firmus* I‐201582	2013	4‐8‐2010	12‐7‐2011	13‐7‐2011	16‐8‐2012	17‐8‐2012	15‐3‐2013	1154	Regulation No. 1107/2009
*Streptomyces lydicus* WYEC 108	2015	6‐8‐2010	4‐5‐2012	5‐5‐2012	14‐10‐2013	15‐10‐2013	14‐7‐2014	1609	Regulation No. 1107/2009
*Bacillus amyloliquefaciens* subsp. *plantarum* D747	2015	21‐10‐2010	14‐1‐2013	15‐1‐2013	27‐3‐2014	28‐3‐2014	10‐10‐2014	1623	Regulation No. 1107/2009
*Bacillus pumilus* QST 2808	2014	3‐12‐2010	8‐5‐2012	9‐5‐2012	25‐7‐2013	26‐7‐2013	20‐3‐2014	1368	Regulation No. 1107/2009
Pepino mosaic virus strain CH2 isolate 1906	2015	30‐7‐2012	8‐1‐2014	9‐1‐2014	18‐12‐2014	19‐12‐2014	29‐4‐2015	1103	Regulation No. 1107/2009
*Trichoderma atroviride* strain SC1	2016	6‐11‐2012	27‐5‐2014	28‐5‐2014	20‐4‐2015	21‐4‐2015	19‐5‐2016	1338	Regulation No. 1107/2009
*Beauveria bassiana* strain 147	2017	6‐11‐2012	2‐10‐2014	3‐10‐2014	3‐12‐2016	4‐12‐2016	23‐3‐2017	1673	Regulation No. 1107/2009
*Beauveria bassiana* strain NPP111B005	2017	6‐11‐2012	7‐10‐2014	8‐10‐2014	6‐12‐2016	7‐12‐2016	23‐3‐2017	1674	Regulation No. 1107/2009
*Saccharomyces cerevisiae* strain LAS02	2016	9‐3‐2013	4‐12‐2014	5‐12‐2014	18‐12‐2015	19‐12‐2015	19‐4‐2016	1215	Regulation No. 1107/2009
*Bacillus amyloliquefaciens* MBI 600	2016	28‐6‐2013	5‐1‐2015	6‐1‐2015	4‐12‐2015	5‐12‐2015	12‐7‐2016	1176	Regulation No. 1107/2009
*Bacillus amyloliquefaciens* strain FZB24	2017	19‐6‐2013	13‐4‐2015	14‐4‐2015	6‐10‐2016	7‐10‐2016	23‐3‐2017	1442	Regulation No. 1107/2009
Mild Pepino Mosaic Virus isolate VC 1	2017	2‐12‐2013	10‐11‐2015	11‐11‐2015	6‐12‐2016	7‐12‐2016	24‐1‐2017	1213	Regulation No. 1107/2009
Mild Pepino Mosaic Virus isolate VX 1	2017	2‐12‐2013	10‐11‐2015	11‐11‐2015	6‐12‐2016	7‐12‐2016	24‐1‐2017	1213	Regulation No. 1107/2009

## Appendix

**Table:** **Appendix B.** Overview of the considered active substances in the US within the reference period (2000–2017)

Active substance	Year first registered	Submission date	Notice of application	Closing of comments	Final decision	T	Regulatory framework
*Pseudomonas chlororaphis* strain 63‐28	2001	20‐11‐1998			21‐12‐2001	1127	Pre‐PRIA
*Bacillus subtilis* var. *amyloliquefaciens* strain FZB24	2000	8‐2‐1999			20‐1‐2000	346	Pre‐PRIA
*Trichoderma harzianum* Rifai strain T‐22	2000	7‐4‐1999			14‐6‐2000	434	Pre‐PRIA
*Chondrostereum purptireum* isolate PFC 2139	2004	15‐4‐1999			20‐9‐2004	1985	Pre‐PRIA
*Metarhizium anisopliae* strain 52	2003	28‐5‐1999			6‐6‐2003	1470	Pre‐PRIA
QST 713 strain of *Bacillus subtilis*	2000	16‐6‐1999			30‐8‐2000	441	Pre‐PRIA
*Coniothyrium minitans* strain CON/M/91‐2008	2001	1‐7‐1999	24‐6‐2000	24‐7‐2000	1‐3‐2001	609	Pre‐PRIA
Indian Meal Moth Granulosis Virus	2001	7‐3‐2000	31‐8‐2001	30‐9‐2001	21‐12‐2001	654	Pre‐PRIA
Bacteriophage active against *Xanthomonas campestris* pv. vesicatoria and *Pseudomonas syringae* pv. Tomato	2005	19‐4‐2000			9‐12‐2005	2060	Pre‐PRIA
*Streptomyces lydicus* strain WYEC 108	2004	27‐4‐2000	1‐8‐2000	1‐9‐2000	24‐5‐2004	1488	Pre‐PRIA
*Bacillus pumilus* strain QST 2808	2004	31‐5‐2000	8‐5‐2002	8‐6‐2002	3‐11‐2004	1617	Pre‐PRIA
*Alternaria destruens* atrain 059	2005	7‐7‐2000			5‐5‐2005	1763	Pre‐PRIA
*Pseudozyma flocculosa* strain PF‐A22 UL	2002	4‐10‐2000			27‐9‐2002	723	Pre‐PRIA
*Bacillus licheniformis* SB3086	2003	29‐12‐2000	26‐6‐2002	26‐7‐2002	4‐2‐2003	767	Pre‐PRIA
*Bacillus pumilus* strain GB34	2001	7‐5‐2001	31‐12‐2001	30‐1‐2002	13‐3‐2003	675	Pre‐PRIA
*Beauveria bassiana* strain 447	2002	19‐9‐2001			1‐9‐2002	347	Pre‐PRIA
*Puccinia thlaspeos* strain woad (dyer's woad rust)	2002	14‐11‐2001	8‐3‐2002	8‐4‐2002	6‐6‐2002	204	Pre‐PRIA
*Verticillium* isolate WCS850	2005	1‐3‐2002			19‐10‐2005	1328	Pre‐PRIA
*Beauveria bassiana* HF23	2006	1‐3‐2002	7‐12‐2005	6‐1‐2006	27‐12‐2006	1762	Pre‐PRIA
*Chondrostereum purpureum* strain HQ1	2005	3‐9‐2002	24‐12‐2003	23‐1‐2004	3‐6‐2005	1004	Pre‐PRIA
*Aspergillus flavus* strain AF36	2003	14‐2‐2003	12‐3‐2003	3‐7‐2003	23‐7‐2003	159	Pre‐PRIA
*Paecilomyces lilacinus* strain 25 1	2005	14‐11‐2003	14‐11‐2003	14‐12‐2003	30‐3‐2005	502	Pre‐PRIA
*Asvereillus flavusem* NRRL 21882	2004	20‐1‐2004	14‐4‐2004	14‐5‐2004	28‐5‐2004	129	PRIA 1
*Muscodor albus* QST 20799	2005	4‐7‐2004			22‐2‐2006	598	PRIA 1
*Chenopodium ambrosioides* var. *ambrosioides*	2008	25‐2‐2005	18‐5‐2005	18‐7‐2005	16‐4‐2008	1146	PRIA 1
*Pythium oligandrum* DV 74	2007	25‐5‐2005	27‐5‐2005	27‐7‐2005	7‐5‐2007	712	PRIA 1
*Pantoea agglomerans* strain E325; NRRL B‐21856	2006	22‐6‐2005			11‐9‐2006	446	PRIA 1
Zucchini Yellow Mosaic Virus ‐ Weak Strain	2007	28‐2‐2006			6‐8‐2007	524	PRIA 1
*Colletotrichum gloeosporioides* f. sp *aeschynomene* and fermentation medium	2006	8‐3‐2006			28‐4‐2006	51	PRIA 1
*Trichoderma hamatumisolate* 382	2010	20‐2‐2007	22‐7‐2009	22‐8‐2009	13‐7‐2010	1239	PRIA 1
*Candida oleophila* strain 0	2009	28‐12‐2007		28‐3‐2008	13‐5‐2009	502	PRIA 1
*Trichoderma asperellum* (ICC 012)	2010	8‐2‐2008	29‐10‐2008	29‐12‐2008	4‐3‐2010	755	PRIA 2
*Trichoderma gamsii* (ICC 080)	2010	8‐2‐2008	29‐10‐2008	29‐12‐2008	4‐3‐2010	755	PRIA 2
*Pasteuria usgae* ‐ BL1	2009	5‐5‐2008	13‐8‐2008	13‐10‐2008	2‐6‐2009	393	PRIA 2
*Pseudomonoa fluorescens* CL145	2011	3‐12‐2008	16‐3‐2009	16‐4‐2009	29‐7‐2011	968	PRIA 2
*Isaria fumosorosea* strain FE 9901	2011	1‐5‐2009	10‐5‐2010	10‐6‐2010	8‐5‐2011	737	PRIA 2
*Bacillus subtilis* strain CX‐9060	2011	30‐7‐2009	10‐3‐2010	10‐4‐2010	15‐12‐2011	868	PRIA 2
*Aureobasidium pullulans* strain DSM 14941	2012	18‐9‐2009	10‐3‐2010	10‐4‐2010	31‐1‐2012	865	PRIA 2
*Aureobasidium pullulans* strain DSM 14940	2012	18‐9‐2009	10‐3‐2010	10‐4‐2010	31‐1‐2012	865	PRIA 2
*Trichoderma virens* strain G‐41	2012	18‐9‐2009	10‐3‐2010	9‐4‐2010	6‐2‐2012	871	PRIA 2
*Chromobacterium subtsugae* strain PRAA4‐1T	2011	22‐12‐2009	3‐3‐2010	3‐4‐2010	27‐9‐2011	644	PRIA 2
*Bacillus thuringiensis* subspecies *galleriea*, strain SDS‐502, fermentation solids, spores and insecticidal toxins	2013	1‐7‐2011	10‐3‐2010	10‐4‐2010	6‐6‐2013	706	PRIA 2
*Pasteuna* spp. (Rotylenchulusremformisnematode)‐Pr3	2012	1‐7‐2010	24‐11‐2010	24‐12‐2010	26‐7‐2012	756	PRIA 2
*Pasteuria nishizawae* – Pn1	2012	1‐7‐2010	24‐11‐2010	27‐12‐2010	28‐2‐2012	607	PRIA 2
*Bacillus amyloliquefaciens* strain D747	2011	26‐7‐2010	2‐2‐2011	2‐3‐2011	8‐12‐2011	500	PRIA 2
Heat‐killed *Burkholderia* spp. strain A396 cells and spent fermentation media	2014	1‐8‐2010	2‐2‐2011	3‐3‐2011	28‐2‐2014	1307	PRIA 2
*Bacillus thuringiensis* subsp. *kurstaki*, strain VBTS‐2546	2012	1‐8‐2011	12‐10‐2011	12‐11‐2011	4‐9‐2012	400	PRIA 2
*Bacillus pumilus* strain BU F‐33	2013	1‐1‐2012	27‐6‐2012	27‐7‐2012	12‐6‐2013	528	PRIA 2
*Helicoverpa zea* ABA Nucleopolyhedrovirus‐U	2014	1‐9‐2012	12‐3‐2013	20‐5‐2013	5‐3‐2014	550	PRIA 2
*Pseudomonas fluorescens*, strain D7	2014	5‐10‐2012			28‐8‐2014	692	PRIA 3
*Beauveria bassiana* strain ANT‐03	2014	25‐3‐2013	11‐12‐2013	10‐1‐2014	30‐3‐2015	735	PRIA 3
*Bacillus subtilis* strain IAB/BS03	2015	7‐5‐2013	21‐4‐2015	20‐2‐2015	5‐2‐2015	639	PRIA 3
*Helicoverpa armigera* nucleopolyhedrovirus strain BV‐0003	2015	25‐10‐2013	18‐4‐2014	19‐5‐2014	3‐11‐2015	739	PRIA 3
*Spodoptera exigua* multinucleopolyhedrovirus (SeMNPV) strain BV‐0004	2015	25‐10‐2013	18‐4‐2014	19‐5‐2014	2‐12‐2015	768	PRIA 3
*Bacillus mycoides* isolate J	2016	5‐3‐2014	4‐2‐2015	6‐3‐2015	3‐10‐2016	943	PRIA 3
*Muscodor albus* strain SA‐2013	2016	21‐4‐2014	21‐1‐2015	20‐2‐2015	15‐11‐2016	939	PRIA 3
*Bacillus amyloliquefaciens* strain PTA‐4838	2016	24‐9‐2014	20‐7‐2015	19‐8‐2015	24‐6‐2016	639	PRIA 3
*Bacillus thuringiensis* ssp. *kurstaki* strain EVB‐113‐19	2016	14‐10‐2014	20‐7‐2015	19‐5‐2015	16‐6‐2016	611	PRIA 3
*Spodoptera frugiperda* MNPV‐3AP2	2016	6‐5‐2015	18‐5‐2015	24‐9‐2015	24‐10‐2016	537	PRIA 3
*Phlebiopsis gigantea* strain VRA 1992	2016	12‐5‐2015	5‐8‐2015	4‐9‐2015	18‐7‐2016	433	PRIA 3
*Bacillus thuringiensis* subsp. *israelensis*, strain SUM‐6218	2016	1‐6‐2015	4‐4‐2016	4‐5‐2016	9‐11‐2016	527	PRIA 3
*Bacillus thuringiensis* subsp. *tenebrionis* strain SA‐10	2016	16‐6‐2015	17‐12‐2015	19‐1‐2016	28‐10‐2016	500	PRIA 3
*Pseudomonas chlororaphis* strain AFS009	2017	2‐10‐2015	18‐5‐2016	24‐6‐2016	23‐6‐2017	630	PRIA 3
Registration cases with missing data (not considered for time span analysis)							
*Bacillus sphaericus* 2362, serotype H5a5b, strain ABTS 1743	2000						Pre‐PRIA
*Cydiapomonella granuloviru*s	2000						Pre‐PRIA
*Bacillus thuringiensis* subsp. kurstaki strain EG7841 Lepidopteran active toxin	2002				4‐9‐2002		Pre‐PRIA
*Bacillus thuringiensis* subsp. aizawai strain NB200	2005		19‐9‐2001		10‐6‐2005		PRIA 1
Bacteriophage active against *Zanthomonas campestris* pv. vesicatoria	2005				9‐12‐2005		PRIA 1
*Pantoea agglomerans* strain C‐9‐1	2006				8‐9‐2006		PRIA 2
*Bacillus firmus* strain I‐201582	2008		7‐3‐2007	7‐4‐2007	28‐4‐2008		PRIA 2
*Ulocladium oudemansii* (U3 Strain)	2009		29‐10‐2008	29‐11‐2008	16‐10‐2009		PRIA 2
Brewer's yeast extract hydrolysate from *Saccharomyces cerevisiae*	2004		6‐8‐2003	6‐9‐2003	2‐2‐2004		PRIA 1
*Bacillus thuringiensis* subsp. *kurstaki* strain EG7841 Lepidopteran active toxin	2002				4‐9‐2004		Pre‐PRIA
Dried fermentation solids and solubles of *Myrothecium verrucaria*	2000				27‐4‐2000		Pre‐PRIA
